# Dissociation method influences immune cell composition in porcine tonsil cell suspensions

**DOI:** 10.3389/fimmu.2026.1896948

**Published:** 2026-08-13

**Authors:** Luise Hohensee, Alexa Eisenmann, Emma L. Lemberger, Xin Qu, Tanja Groll, Katja Steiger, Friederike Ebner

**Affiliations:** 1 Chair of Infection Pathogenesis, Technical University of Munich, Freising, Germany; 2 Comparative Experimental Pathology, Technical University of Munich, München, Germany

**Keywords:** dissociation, germinal center, pig, storage, T follicular helper cells, tonsil

## Abstract

**Introduction:**

Porcine tonsils are follicle-rich secondary lymphoid organs positioned at the oropharyngeal interface. In pigs, a major livestock species, they serve as a primary barrier against inhaled and ingested pathogens and play a central role in mucosal immunity. Despite their importance as a point of entry and as a reservoir for numerous pathogens, the cellular landscape driving tonsillar immune responses in pigs remains insufficiently defined.

**Methods:**

As a first step toward improving our understanding of tonsil cellularity and enabling advanced *in vitro* models, we evaluated how different tissue dissociation methods affect the relative frequencies of both, abundant and rare innate and adaptive immune cell subsets from swine tonsils. We investigated the influence of mechanical vs. enzymatic dissociation methods on the isolation of tonsillar leukocytes, particularly those involved in the GC reaction: T follicular helper cells (Tfh), germinal center (GC) B cells and plasma cells, as well as myeloid subsets.

**Results:**

Mechanical dissociation increased total cell yield by 27%, while enzymatic digestion improved cell viability by 21%. During short-term storage immune cell frequencies decreased over time, with mechanical dissociation detecting higher cell numbers and enzymatic digestion providing higher viability. Mechanical isolation improved detection of CD172α+ myeloid cells and Blimp-1+IRF-4+ plasma B cells, whereas enzymatic digestion increased relative frequencies of CD79α+ B cells and ICOS+Bcl6+ T follicular helper cells (Tfh). Surprisingly, enzymatically isolated cells showed impaired cytokine production compared to mechanically isolated cells and presented reduced viability in 3D hanging drop cultures.

**Discussion:**

These findings indicate that mechanically isolated tonsillar cells may be preferentially suited for spheroid cultures. However, to establish a robust 3D culture system modelling GC reaction, further optimization is needed. Our study highlights the importance of tailoring tissue dissociation strategy to specific experimental endpoints.

## 1 Introduction

**Figure 1:**
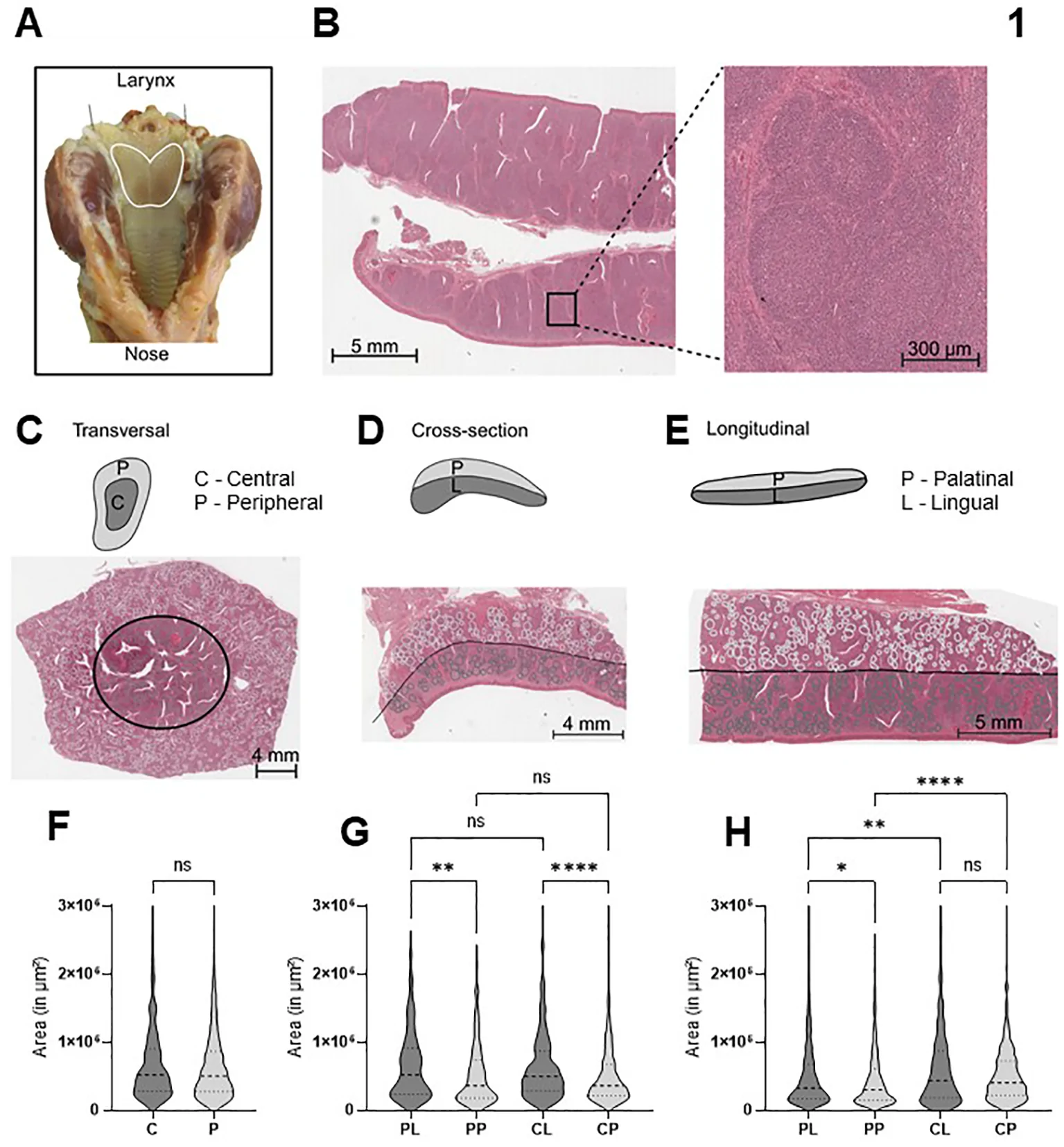
Location influences follicle size within the porcine soft palatine tonsil. **(A)** The soft palatine tonsil is a paired lymphoid structure located at the transition from the hard to the soft palate, centrally between the two maxillary bones. **(B)** Hematoxylin eosin (HE)-stained sections of the soft palatine tonsil reveal a thick epithelial layer on the lingual side and prominent follicle structures. **(C–H)** HE stainings were evaluated to determine how central (C) or peripheral (P) as well as lingual (L) or palatinal (P) localization impact follicle size in transversal cuts **(C, F)**, cross section **(D, G)** and longitudinal cuts **(E, H)** of the soft palatine tonsil. Per orientation the tonsil of one animal was analyzed. The area of at least 150 follicles per localization was measured (technical replicates). Statistics were performed as Mann-Whitney test **(F)** or Kruskal-Wallis-Test **(G, H)**. Significant differences were depicted according to the P-value as non-significant (ns) (P > 0.05), *(P ≤ 0.05), **(P ≤ 0.01), ****(P ≤ 0.0001).

Tonsils are prominent lymphoid structures located at the pharynx. They act as a first line of defense against inhaled or ingested pathogens. In pigs, five tonsils can be distinguished: The lingual tonsil at the base of the tongue, the paraepiglottic tonsils at the base of the epiglottis, the pharyngeal tonsil at the roof of the nasopharynx, the tubal tonsils located in the tuba auditiva, and the tonsil of the soft palate. This two-lobed flat structure at the roof of the oropharynx is especially well developed in swine, measuring approximately 5 cm x 3 cm per lobe in 6–8-month-old Landrace breeds (**Figure 1A**) (1). The tonsil of the soft palate is directly exposed to ingesta. Therefore, the lingual side is lined with thick epithelia to protect against mechanical damage, while the palatine side is covered by loose connective tissue.

Due to their exposed location, tonsils are not only a first point of contact for food- and airborne antigens and pathogens but can also serve as primary sites of replication and chronic persistence for many relevant livestock pathogens. Porcine circovirus type 2, African swine fever virus, and *Streptococcus suis* have been observed to replicate in the tonsils, while classical swine fever virus, porcine reproductive and respiratory syndrome virus, and foot-and-mouth disease virus have been shown to persist in tonsils (2–7). Moreover, food−relevant pathogens such as *Salmonella* spp. and *Yersinia enterocolitica* have been detected in porcine tonsils (8, 9). Their presence underscores the importance of the porcine tonsil as a target organ for research, both in veterinary medicine and in the context of zoonotic disease transmission.

Recent studies have introduced human tonsil-derived complex cell culture systems as *ex vivo* platforms to test for the immunogenicity of vaccines and to model germinal center reactions (GCR) (10, 11). Such approaches are currently lacking for livestock species, but their implementation is urgently warranted, particularly in light of the pressing need to advance 3R-complient methodologies and to address the persistent underrepresentation of livestock-focused alternative models.

Germinal centers (GC) are areas of B cell maturation and clonal expansion take place (12, 13). Due to their exposed location at the oronasal route porcine tonsils are a key site for antigen sampling and immune cell activation during infection and vaccination (7, 14, 15). Within lymphoid follicles the GC are formed. During the GC reaction, following antigen presentation by follicular dendritic cells (FDC), GC B cells, orchestrated by T follicular helper cells (Tfh), undergo maturation and expansion processes (12, 13, 16–18).

While definitive proof is not available, studies analyzing the porcine lymphoid tissues during infection (19) and investigating the porcine immune cell expression profile suggest GC-like responses in pigs (20). Similar to other mammals, the antagonistic interaction between the transcription factors Bcl6 and Blimp-1 shapes and controls B cell differentiation during the GC reaction in swine (21–23). Furthermore, histological analyses of porcine tonsils identified follicular structures likely containing FDC networks (24). This indicates, that porcine FDCs have a similar role of antigen presentation and retention for affinity-based selection as human or murine FDCs (12, 25). Recently, porcine Tfh cells have been identified as CD4+ Bcl6+ ICOS+ T cells (26), further indicating that GC processes are conserved among mammalian species. However, functional details or mechanistic insights into the porcine GC response, specifically in tonsils, are limited.

In human tonsil-derived 3D-cultures, mechanical dissociation is most common (10, 11), but perfusion and explant models are also used (27, 28). However, enzymatic digestion may be beneficial for isolating rare cell types, such as Tfh or myeloid cells. To our knowledge, no systematic comparison of mechanical and enzymatic dissociation of tonsillar tissue has been reported. Therefore, we evaluated both approaches for the isolation of porcine tonsillar immune cells.

Due to their exposed location and their function as an interface between mucosal and systemic immunity, pig-specific, tonsil-derived complex cell culture systems might be best suited to investigate tonsil-specific immune responses and to accurately model the porcine GCR *ex vivo*. To establish such culture systems, firstly isolation methods are needed that reliably recover GCB cells, rare Tfh cells and antigen presenting cells (APC). In this study, we therefore evaluated how tissue dissociation methods influence both the cellular composition of porcine tonsillar immune cells and their functional properties, including cytokine responsiveness. Special emphasis was placed on cells, that are crucial for modelling the GCR, such as B cells, Tfh and antigen-presenting cells. By comparing dissociation strategies, we aim to identify methods that enrich relevant cell populations and support functionality best, thereby providing a suitable foundation for subsequent establishment of tonsil-derived 3D- long-term cultures.

## 2 Materials and methods

### 2.1 Biological materials

Animal tissues were collected from healthy, freshly slaughtered adult pigs (at least 6 months old, 120 kg) at the SBL slaughterhouse, Landshut (Germany). Slaughtered pigs in Germany are typically females or castrated males of the commercial crossbreed of German Landrace, Large White and Pietrain. Samples were transported (~1h) in cold RPMI-1640 (R8758, Merck, Germany) supplemented with 2% Penicillin/Streptomycin (P/S; P4333, Merck, Germany) and 1% Fetal Bovine Serum (FBS; S0615, Merck, Germany) and stored at 4 °C until further use. To document the anatomical location of the tonsil within the skull (**Figure 1A**), a specimen was prepared from an animal originating from the TUM Animal Research Center during planned necropsies (license number ROB-55.2-2532_Vet_02-23-82).

### 2.2 Histological imaging of tonsillar tissue

To evaluate tonsillar follicle distribution and density, hematoxylin and eosin (H&E) staining was performed following standard protocols. Briefly, tonsil samples were rinsed with PBS directly after collection and then transferred into 4% paraformaldehyde (10160052, Fisher scientific, USA) for fixation. After fixation and cutting, CEP further processed the samples and performed H&E staining. Representative images were taken and analyzed using Aperio ImageScope x64 (v12.4.6.5003, Leica Biosystems, Wetzlar, Germany).

Additionally, immunofluorescence microscopy was performed on O.C.T. (16-004004, Sakura, USA) embedded frozen samples. Freshly collected samples were cut to size and placed in 15 x15 x15mm cryomolds and subsequently snap-frozen at -20 °C. Solidified samples, were sectioned at 14 µm using a Microm cryotome (ThermoFisher, USA) and mounted onto positively charged slides. After air-drying and washing with PBS to remove residual O.C.T. sections were fixed in 99% ice-cold acetone and again washed twice with PBS. Subsequently, blocking and staining were performed with volumes of 100 µL per sample within hydrophobic barriers. For blocking samples were incubated with UltraCruz^®^ Blocking Reagent (sc-516214, Santa Cruz Biotechnology, USA) for 30 min at room temperature. Staining with primary antibodies was carried out overnight at 4 °C, secondary antibodies were incubated for 1h at room temperature. The antibodies used for immune fluorescence microscopy (IF) are listed in **Table 1**. Between staining steps, slides were washed with PBS to remove unbound antibodies. Finally, anti-fade mounting medium with DAPI (H-1200, Vector Laboratories, USA) was added and the slides were covered with cover slides. Prepared samples were scanned using the ECHO Revolve Microscope (Discover Echo Inc., USA). Single optical sections were acquired for each fluorescence channel and subsequently merged using ImageJ.JS (NIH, USA). Image post-processing was performed in ImageJ.JS using identical parameters for all images.

**Table 1:** Antibodies used in immunofluorescence imaging of O.C.T. embedded tonsillar tissue.

Antibody	Clone	Isotype	Dilution	Company
Mouse anti-human CD21 unlabeled	B-ly4	Mouse IgG1, κ	1:100	BD Pharmingen™
Mouse anti-pig CD4 unlabeled	74-12-4	Mouse IgG2b, κ	1:50	Thermo Fisher Scientific Inc.
Goat anti-mouse IgG1 Cross Adsorbed Secondary Antibody Alexa Fluor™ 594		Goat, IgG gamma 1	1:500	Thermo Fisher Scientific Inc.
Goat anti-mouse IgG2b Cross Adsorbed Secondary Antibody Alexa Fluor™ 488		Goat/IgG	1:250	Thermo Fisher Scientific Inc.

### 2.3 Tissue dissociation

To compare tissue dissociation methods, porcine tonsils of the soft palate were cleaned by removing surrounding tissues (e.g. connective tissue, fat, muscle). Taking our own data on follicle size into account, samples were collected from central regions of the prepared tonsils and represented a complete cross-section from the lingual to the palatinal side. Each sample was then bisected and processed in a paired setting, with one half subjected to mechanical dissociation and the other half to enzymatic digestion. Prior to processing, all samples were weighed and cell yields were subsequently normalized to tissue weight.

For mechanical isolation (M) the tonsil pieces were cut transversely and placed cut side down on 1 mm metal strainers submerged in 10 mL of RPMI supplemented with 1% FBS (wRPMI). Samples were further macerated through the strainer and collected in an additional 20 mL of wRPMI. After centrifugation (10 min, 350 xg, 10 °C) the supernatant was removed and the pellet was resuspended in 10 mL complete media (10% FBS, 2% P/S, 1% Amphotericin B). To remove debris, the suspension was passed through a 100 µm strainer (732-2759, vwr, USA). Afterwards, strainers were rinsed with an additional 10 mL to remove remaining cells. Cell number and viability were assessed after trypan blue (T8154, Sigma Aldrich (Merck), Germany) staining for dead cell exclusion using the Countess 3 automatic cell counter (Invitrogen by ThermoFisher Scientific, USA).

Based on several pre-optimization steps concerning the composition of the enzyme cocktail and pre-cutting procedures (**Supplementary Figure 1**), we selected a digestion mix containing 1mg/mL Collagenase IV (C4-22, Sigma Aldrich (Merck), Germany), 0.18 mg/mL DNAse I (DN25, Sigma Aldrich (Merck), Germany), 0.125 mg/mL Liberase DH (5401054001, Roche, Switzerland) and 0.125 mg/mL Liberase TM (5401119001, Roche, Switzerland) in serum-free RPMI-1640 for enzymatic digestion (D). Tonsils were sliced into approximately 1mm slices and macerated with sharp scissors to optimize digestion efficiency. 1 mL of digestion mix per g of tonsil tissue was added. Subsequently, the samples were incubated in a shaking water bath (37 °C, 250 rpm) for 1h. To stop digestion, samples were filtered through a 100 µm strainer and thoroughly washed with complete, FBS-containing RPMI media. After centrifugation (10 min, 350 xg, 10 °C), cells were resuspended in complete media and counted using trypan blue staining.

### 2.4 Short-term storage

Future biobanking endeavors require that tissue can be safely transported over long distances. To analyze the impact of short-term (1–2 days) storage during shipping, tonsils were placed in RPMI containing 2% P/S and 1% FBS and kept at 4 °C. After 24 and 48 hours, tonsil pieces were dissociated according to the described protocols. Cell numbers and viability were similarly assessed.

### 2.5 Cell staining and flow cytometry

For flow cytometrical staining, cells were plated at a density of 3 million cells per well in V-bottom 96-well plates (821.583, Sarstedt, Germany). Cells were washed and resuspended in FACS buffer (PBS + 2% FCS + 1 mM EDTA). Fc blocking was routinely carried out using FACS buffer containing heat-inactivated mouse-serum (in house, 1:500) for 5 min, except for B cell staining where PBS supplied with 2% heat-inactivated pig serum (in house) and 1 mM EDTA was used for blocking, surface staining and washing.

All surface staining, including viability staining, was performed for 15 min at 4 °C. Antibodies were prepared in FACS buffer at a staining volume of 30µL per sample. For intracellular staining, the fixation buffer (420801, Biolegend, USA) was used according to manufacturer’s instructions, intranuclear fixation was achieved with Foxp3/Transcription Factor Staining Buffer Set (00-5523-00, Invitrogen by ThermoFisher, USA). Antibodies targeting intracellular or intranuclear epitopes were incubated for 30 min at room temperature in the dark. Samples were acquired using the Attune NxT Flow Cytometer (ThermoFisher, USA) and analyzed with FlowJo (Version 10, FlowJo LLC, USA).

All antibodies and dyes used in this study are indicated in **Table 2**. All antibodies had previously been reported to recognize porcine markers (26, 29, 30). FMOs and single stain controls were used for analyzing (**Supplementary Figures 2**–**4**).

**Table 2:** Antibodies and dyes used in flow cytometry.

Antigen	Clone	Isotype	Fluorophore	Company	Article no
Bcl6	K112-91	mouse IgG1k	PE	BD	561522
Blimp-1	3H2E8	mouse IgG1k	AF488	SantaCruz	sc-66015 AF488
CADM1	3E1	chicken IgY	unlab	MBL	cm004-6
CD3	PPT3	mouse IgG1	unlab PB Biotin	BioRad BioRad Novus	MCA5951GA MCA5951PB NBP1-28227
CD3e	BB-23-8E6-8C8	mouse IgG2ak	PerCP-Cy5.5	BD	561478
CD4a	74-12-4	mouse IgG2bk	PE-Cy7 PerCP-Cy5.5	BD	561473 561474
CD8a	76-2-11	mouse IgG2ak	APC	BD	561475
CD8a	PT36B	mouse IgG1	Unlab	Kingfisher (biomol)	ws0618s-100
CD14	TÜK4	mouse IgG2ak	VioBlue	Miltenyi Biotech	130-113-152
CD16	G7	mouse IgG1	AF647	BioRad	MCA1971A647
CD21	B-ly4	mouseIgG1	unlab	BD	555421
CD45RA	MIL13	mouse IgG1	FITC	BioRad	MCA1751F
CD79a	HM57	mouse IgG1ak	PE	Dako	r7159
CD79a	HM47	mouse IgG1ak	PerCP-Cy5.5	ThermoFisher	45-0792-42
CD163	2A10/11	mouse IgG1	FITC	ThermoFisher	MA5-28292
CD169	3B11/11	mouse IgG1	APC	BioRad	MCA2316A647
CD172a	74-22-15A	mouse IgG2bk	FITC PE	BD	561498 561499
ICOS	C398.4A	armenian Hamster IgG	AF700	Biolegend	313528
IFNg	P2G10	mouse IgG1k	PE	BD	559812
IgG1	RMG1-1	rat IgG	APC-Cy7 BV510	Biolegend	406620 406621
IgG2a	R19-15	rat IgG1k	BV711	BD	744533
IgM	K52 1C3	mouseIgG1	unlab	Biorad	MCA637GA
IgY, chicken		rabbit IgG	Texas-Red	Jackson ImmunoResearch	303-585-003
IL-4	MP4-25D2	rat IgG1k	PE-Cy7	Biolegend	500823
IL-17	eBio64DEC17	mouse IgG1k	FITC	ThermoFisher	11-7179-42
IRF-4	IRF4.3E4	rat IgG1k	PE-Cy7	Biolegend	646414
Ki-67	B56	mouse IgG1k	AF700	BD	561277
MHC-II	MSA3	mouseIgG2a	unlab	in house	
Pax-5	1H9	rat IgG2ak	PE	ThermoFisher	12-9918-80
Streptavidin			BV711	BD	563262
T-bet	4B10	mouse IgG1k	BV421	BioLegend	644816
TCRgd	PGBL22A	mouse IgG1	unlab	KingFisher (biomol)	WS0621S-100
TNFa	MAb11	mouse IgG1k	PB	BioLegend	502920
Zombie Aqua				BioLegend	502920

### 2.6 Intracellular cytokine detection

Freshly mechanically or enzymatically isolated cells were resuspended in complete IMDM (10% FBS, 2% P/S, 1% Amphotericin B (A2942, Sigma Aldrich (Merck), Germany) and plated in round-bottom 96-well plates (650180, Greiner Bio-One, Austria) at 3 million cells per well. Plates were incubated at 38.5 °C with 5% CO_2_ and allowed to rest overnight. Cells were then stimulated with 50 ng/mL PMA (P8139, Merck, Germany) and 500 ng/mL Ionomycin (IO634, Merck, Germany) and 1h post stimulation Brefeldin A (00-4506-51, ThermoFisher, USA) and Monensin (00-4505-51, ThermoFisher, USA) at a 1x working concentration (~5 µg/mL) were applied. After 3h of incubation (38.5 °C) cells were harvested, washed with FACS buffer and processed for flow cytometry staining as previously described. For intracellular fixation IC-Fix Buffer (00-8222-49, ThermoFisher, USA) was used according to manufacturer’s instructions.

### 2.7 3D cell cultures

#### 2.7.1 Spheroids in suspension

Mechanically isolated tonsil cells were plated in complete RPMI (10% FBS, 2% P/S, 0.5% Amphotericin B) with a density of 6 million cells per well into 12-well suspension plates (665102, Greiner Bio-One, Austria) and incubated at 38.5 °C, 5% CO_2_. Media was changed every second day by removing and replenishing approx. 30% of the media. For, IF imaging formed spheroids were collected after 6 days of cultivation and carefully washed twice with PBS. Afterwards cells were treated with PBS with 4% PFA (30 min, RT) for fixation and permeabilization. Immunofluorescence staining was performed as previously described (**Table 1**).

#### 2.7.2 Hanging drop cultivation

Tonsil cells were resuspended at a density of 2x10^6^ cells/mL in complete RPMI with 20% Methocoel (M0512, Sigma Aldrich) for gravity-assisted aggregate formation. Cells were then dispensed onto square polystyrene petri dishes (688102, Greiner Bio-One) at 40 µL per drop. Drops, containing approx. 150,000 cells per drop, were placed at a sufficient distance from one another to prevent merging during incubation. Subsequently, plates were flipped and placed on the bottom compartment of the dish, which was prefilled with PBS to reduce evaporation. The now hanging drops were carefully incubated at 37 °C, 5% CO_2_ for up to three days. Viability was assessed daily using trypan blue as previously described. Additionally, morphology of the forming aggregates was assessed by light microscopy (ECHO revolve, Discover Echo Inc., USA). Subsequently, ImageJ was utilized to measure the area of the aggregates and calculate both the Feret’s diameter and the circularity. The Feret’s diameter indicates the longest distance between two points situated on the drawn circumference of the spheroid and serves as an additional size parameter. On the other hand, circularity describes the shape of the aggregate with a value from 1.0 (perfect circle) to 0.0 (angulated, irregular shape).

### 2.8 Statistical analysis

Statistical analyses were performed using GraphPad Prism 10 (GraphPad Software, USA). Where necessary, data were tested for normal distribution (Shapiro-Wilk test and Kolmogorov-Smirnov test). Based on this, appropriate statistical tests were selected: Data concerning follicle size in various regions of the tonsil were isolated by the Mann-Whitney (transversal section) or the Kruskal-Wallis test (longitudinal and cross-section). For all other analyses, paired T-tests (one time point) or 2-way ANOVAs (over time) were applied. Significant differences were depicted according to the P-value as non-significant (ns) (P > 0.05), *(P ≤ 0.05), **(P ≤ 0.01), ***(P ≤ 0.001), ****(P ≤ 0.0001).

## 3 Results

### 3.1 Porcine tonsil microarchitecture

Like other lymphatic structures with direct antigen contact, tonsils are rich in lymphatic follicles (**Figure 1B**). To assess whether the sublocation of tonsillar sampling affects follicular characteristics, follicle size was quantified in central and peripheral regions of both transverse and longitudinal sections, as well as in cross−sections (**Figures 1C–E**). In transverse sections, the central versus peripheral sampling location did not affect follicle size (**Figure 1F**). Likewise, no significant differences in follicle size were observed between central and peripheral regions in cross−sections (**Figure 1G**). In longitudinal sections, however, centrally located follicles were significantly larger than those located in the periphery (**Figure 1H**). In addition, follicles were significantly larger when located on the lingual side of the tonsil compared to the palatine side. This was most apparent in cross-sections (**Figure 1G**). This indicated that centrally located tonsillar tissue, especially from the lingual side, had significantly larger follicle structures than peripheral locations. Therefore, to circumvent a sampling bias, centrally located, complete (lingual through palatine side) cut-outs of the soft palatine were used for all further experiments.

### 3.2 Evaluation of tissue dissociation methods

**Figure 2:**
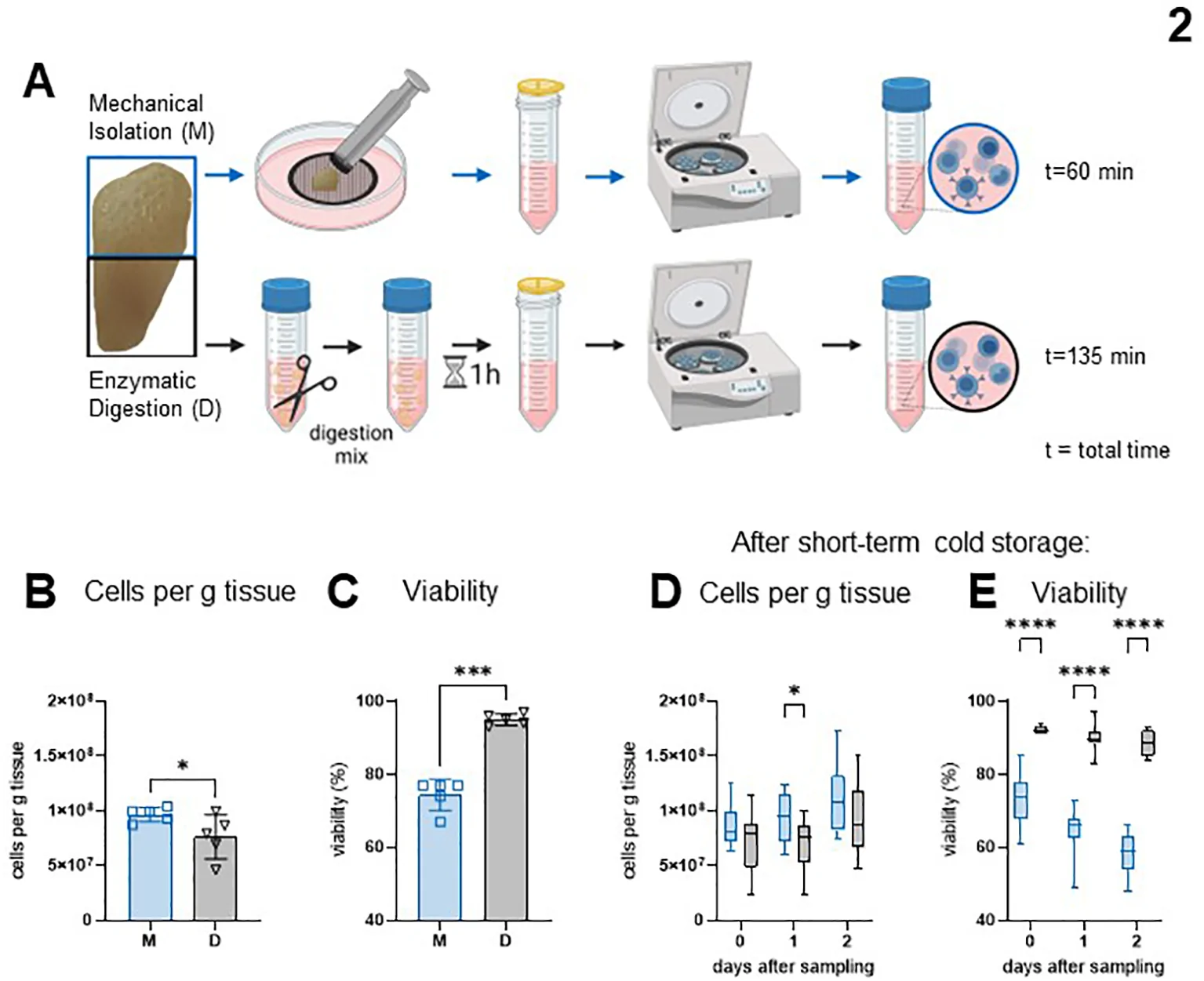
Processing porcine soft palatine tonsils yields large quantities of cells independent of the dissociation method. **(A)** Experimental scheme to isolate tonsillar single cells by mechanical dissociation or enzymatic digestion. **(B–E)** Representative yield and cell viability after mechanical isolation (blue) and enzymatic digestion (grey) on the day of sampling **(B, C)** and after storage at 4 °C for up to 2 days **(D, E)**. Data is from 5 **(B, C)** or 10 **(D, E)** individual pigs is depicted as mean ± SD **(B-C)** or min-max **(D, E)**, mechanical isolation (M, blue), enzymatic digestion (D, black). Statistical analyses were performed as paired t test **(B, C)** or Two-Way ANOVA **(D, E)**. Significant differences were depicted according to the P-value as *(P ≤ 0.05), *** (P ≤ 0.001), ****(P ≤ 0.0001).

Due to their exposed location, tonsils have evolved more fibrously than lymph nodes and, therefore, are more difficult to mechanically process. In a first step, we thus compared mechanical (M) versus enzymatic (D) dissociation methods (**Figure 2A**) in regard to the total number of isolated cells and cell viability. Although both methods consistently yielded high cell numbers, mechanical dissociation produced significantly more cells per gram of tissue than enzymatic dissociation (M: 9.67x10^7^/g ±6.52x10^6^/g; D: 7.64x10^7^/g ±2.01x10^7^/g) (**Figure 2B**). Conversely, enzymatic digestion resulted in significantly higher cell viability (M: 74.4% ± 4.34%; D: 95% ± 1.58%) than mechanical isolation (**Figure 2C**). Short-term storage at 4 °C for 2 days revealed similar differences in recovery: mechanical dissociation consistently yielded higher cell numbers than enzymatic digestion (**Figure 2D**). Although overall viability declined over time, enzymatic isolation reliably produced samples with higher viability compared with mechanical dissociation (**Figure 2E**).

### 3.3 Mechanical isolation increases myeloid cell detection, while digestion enriches B cells

**Figure 3:**
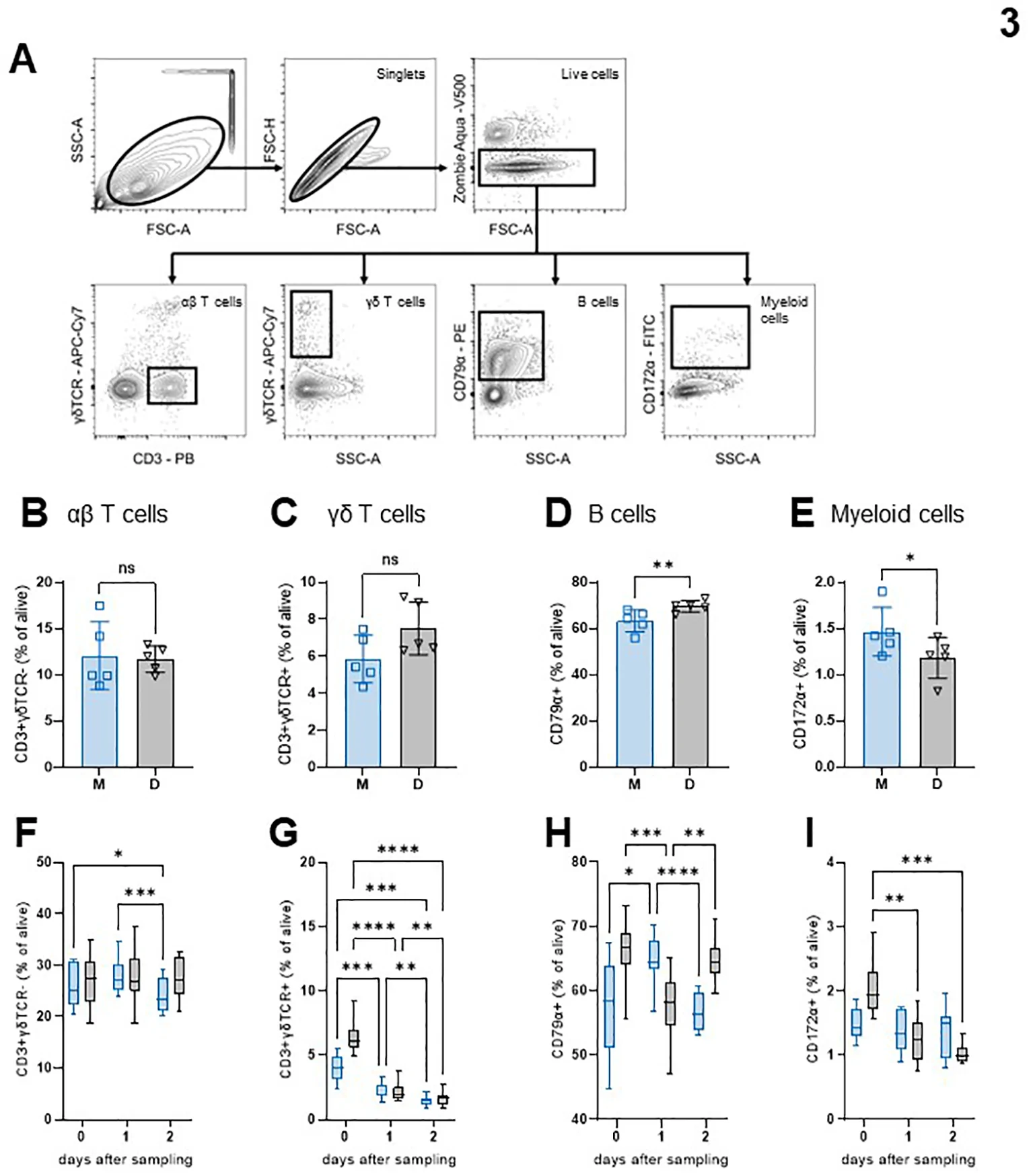
Mechanical isolation ameliorates the relative frequency of myeloid cells (CD172α+), whereas digestion increases the B cell (CD79α+) detection. **(A)** Gating strategy to identify conventional αβ T cells (CD3ϵ+γδTCR-), γδ T cells (CD3ϵ+γδTCR+), B cells (CD79α+) and myeloid cells (CD172α+) from alive, single cells. **(B–E)** Frequency of conventional αβ T cells **(B)**, γδ T cells **(C)**, B cells **(D)** and myeloid cells **(E)** after mechanical (blue) and enzymatic (black) isolation. **(F–I)** Frequency of conventional αβ T cells **(F)**, γδ T cells **(G)**, B cells **(H)** and myeloid cells **(I)** processed by mechanical (blue) or enzymatic (black) isolation after storage at 4 °C for up to 2 days. Data from 5 **(B–E)** or 10 **(F–I)** individual pigs is depicted as mean ± SD **(B–E)** or min-max **(F–I)**. Statistical analyses were performed as paired T-test **(B–E)** or two-way ANOVA **(F–I)**. Significant differences were depicted according to the P-value as non-significant (ns) (P > 0.05), *(P ≤ 0.05), **(P ≤ 0.01), *** (P ≤ 0.001), ****(P ≤ 0.0001).

Utilizing the lineage markers CD3, γδTCR, CD79α and CD172α flow cytometry was performed to provide an overview over major immune cell populations of conventional αβ T cells (CD3+γδTCR-), gd T cells (CD3+γδTCR+), B cells (CD79α+) and myeloid cells (CD172α+) (**Figure 3A**). While no significant differences occurred for conventional αβ T and γδ T cells (**Figures 3B, C**), enzymatic digestion detected significantly more B cells (**Figure 3D**). However, mechanical isolation proved to be beneficial for isolating myeloid cells, as significantly more CD172α+ cells were identified here (**Figure 3E**).

During the 2−day storage period, the frequency of mechanically isolated conventional αβ T cells decreased significantly from day 1 to day 2, whereas the proportion of enzymatically isolated conventional T cells remained stable over time (**Figure 3F**). γδ T cell frequency, however, significantly dropped over time, independent of isolation method used (**Figure 3G**). Although B cell frequencies showed some significant fluctuations over the storage period, they consistently remained high across both isolation methods (>50% of viable cells) (**Figure 3H**). Notably, enzymatic dissociation significantly increased B cell frequencies, even after 2 days of storage. Myeloid cell frequency was also significantly reduced during 2-day storage in both groups (**Figure 3I**). However, higher levels of myeloid cells were recovered in mechanically isolated cells. This indicates that immune cell composition is storage time- and method-dependent with strongest effects on T and myeloid cells.

### 3.4 Enzymatic digestion improves the detection of Bcl6+ T follicular helper cells

**Figure 4:**
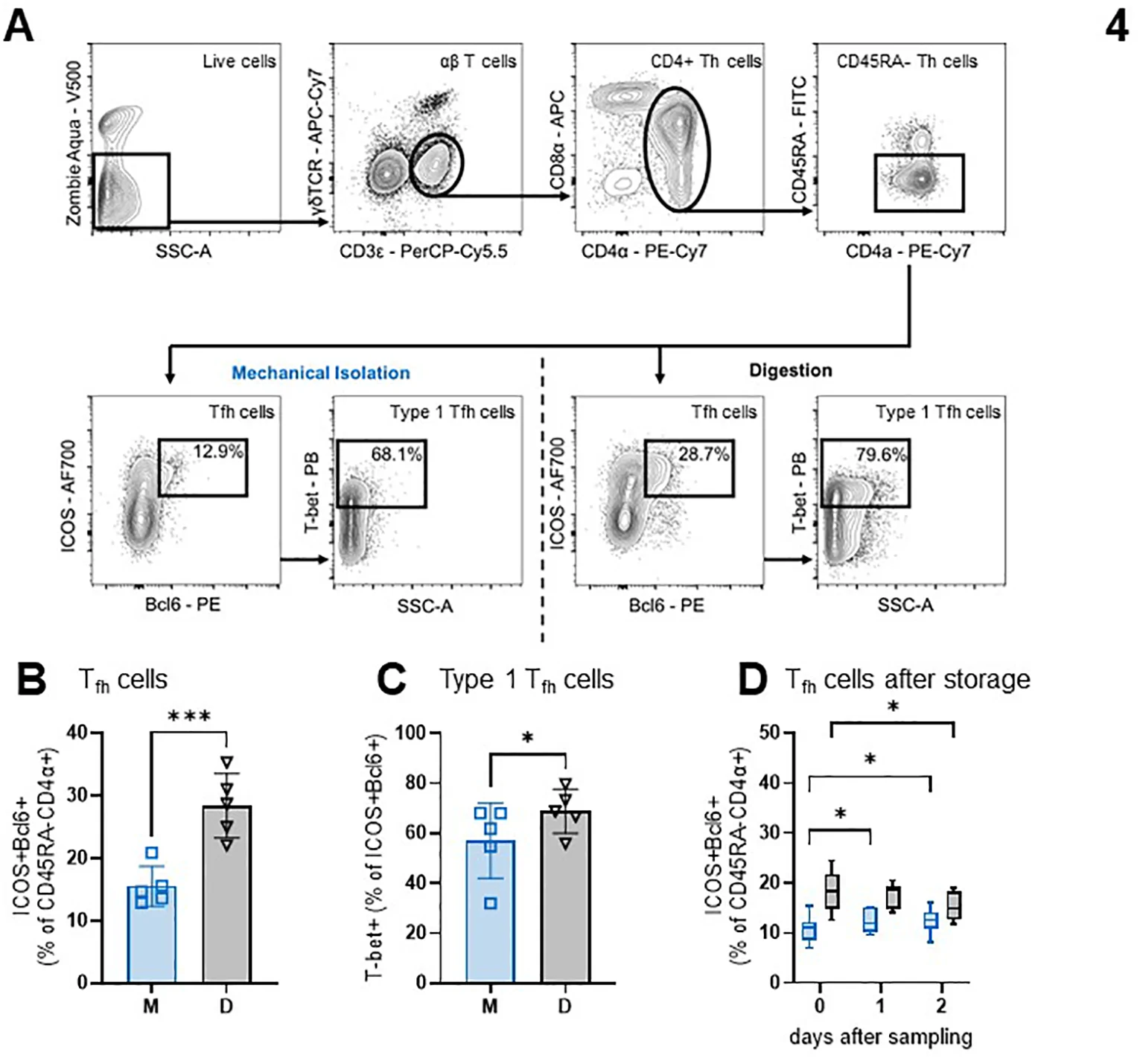
Enzymatic digestion improves isolation of T follicular helper cells. Gating strategy to identify T follicular helper cells (Tfh, Bcl6+ICOS+) and T-bet+ Tfh cells from CD3+γδTCR-CD4α+CD45RA- single cells. Depicted are representative flow cytometrical plots illustrating the Tfh population after mechanical and enzymatic isolation. The T-bet expression profile of Tfh recovered from mechanical and enzymatic dissociation is shown **(A)**. Frequency of Tfh cells **(B)** and T-bet+ Tfh cells **(C)** isolated mechanically (blue) or enzymatically (black). Data from 5 pigs is depicted as mean ± SD. Statistical analyses were performed as paired T-tests. **(D)** Tfh frequency after storage at 4 °C for up to 2 days. Data from 10 individual pigs is depicted as min-max. Statistical analysis was performed as two-way ANOVA. Significant differences were depicted according to the P-valueas non-significant (P>0.05), *(P ≤ 0.05), ***(P ≤ 0.001).

Tfh cells are crucial for B cell activation during the GC reaction. Tfh cells in pigs can be identified as CD3+ γδTCR- CD4+ CD45RA- ICOS+ Bcl6+ population (**Figure 4A**) (26). The transcription factor T-box expressed in T cells (T-bet) can further be used to differentiate type 1 Tfh cells, which are linked to a proinflammatory response, targeting intracellular pathogens.

In our hands, enzymatic digestion yielded significantly enriched Tfh cells compared to mechanical isolation, which was unexpected (M: 15.54% ± 3.17%; D: 28.42% ± 5.14%) (**Figure 4B**). Additionally, 68.84% ± 8.76% T-bet+ Tfh cells were isolated from enzymatically digested samples, compared to 57.02% ± 15.02% from mechanically isolated samples (**Figure 4C**), demonstrating that enzymatic isolation provides a clear advantage for Tfh cell enrichment. Similar to the conventional αβ T cells (**Figure 3F**) Tfh levels remained high after 2-day-storage at 4 °C. Interestingly, while Tfh frequencies decreased by day 2 in the digested samples, Tfh levels in mechanically isolated samples increased over time (**Figure 4D**). This may be related to changes in tissue integrity during storage improving Tfh isolation or a relative enrichment, due to differential survival of other immune cell populations.

### 3.5 Mechanical isolation ameliorates plasma cell and GC B cell detection

**Figure 5:**
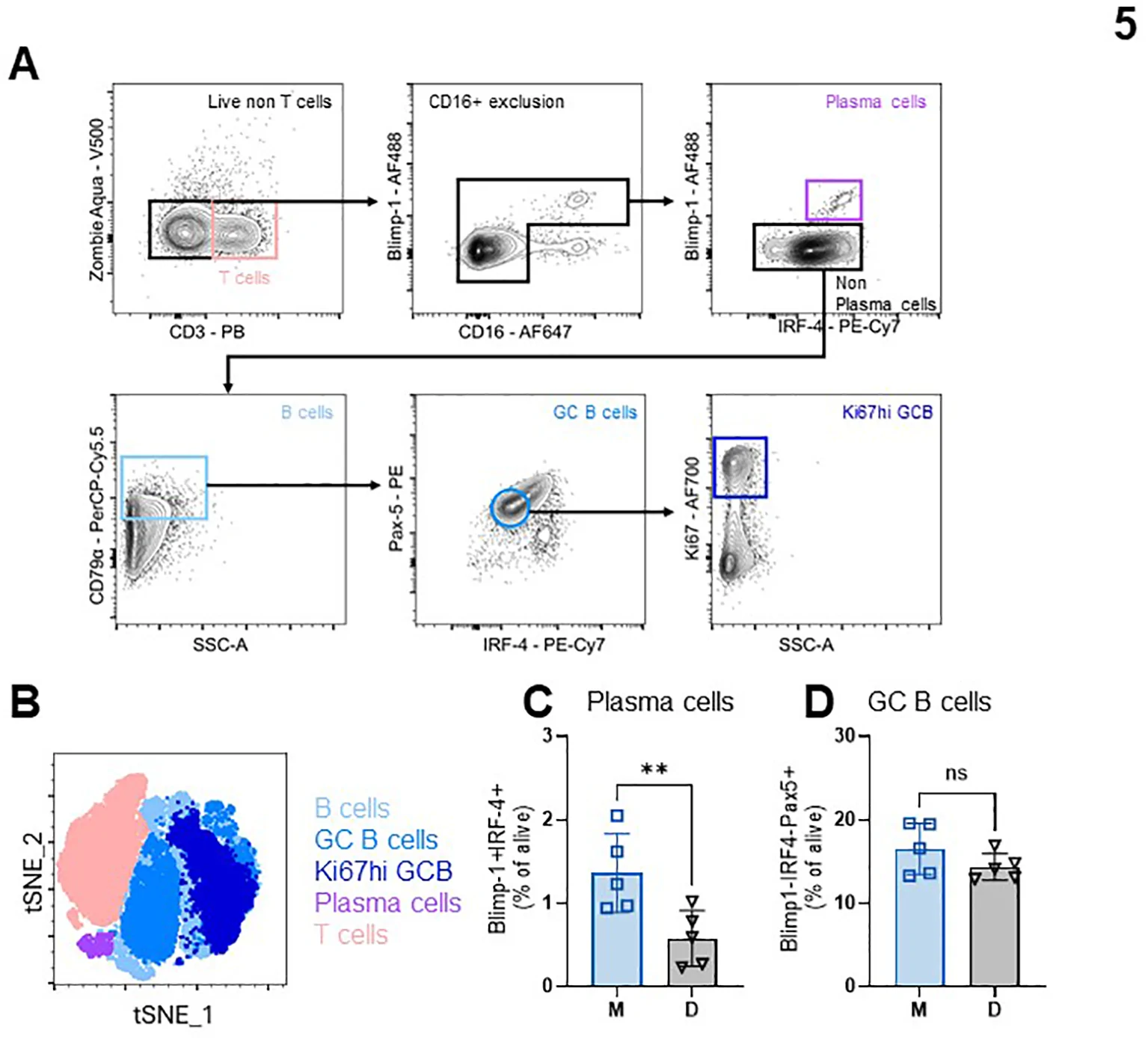
Mechanical isolation increases isolation of plasma cells from tonsillar tissue. **(A)** Gating strategy to identify plasma cells (Blimp-1+IRF-4+) and germinal center (GC) B cells (Blimp-1-IRF-4-Pax-5+) from alive, single cells. **(B)** tSNE analysis of selected B cell subsets. **(C, D)** Tonsil-derived plasma cells **(C)** and GC B cells **(D)** frequencies were analyzed. Data from 5 individual pigs is depicted as mean ± SD, mechanical isolation (M, blue), enzymatic digestion (D, black) are shown. Statistical analyses were performed as a paired t-test. Significant differences were depicted according to the P-valueas non-significant (ns) (P > 0.05), **(P ≤ 0.01).

In addition to Tfh cells, B cells play the central role in the germinal center (GC) reaction. Using the transcription factors Blimp-1, IRF-4 and Pax-5, plasma cells and GC B cells could be clearly distinguished (**Figure 5A**). Non-linear dimension reduction further demonstrated that these populations form well-separated clusters (**Figure 5B**). Within the GC B cell compartment, a distinct subset of highly proliferative Ki67^+^ cells was evident. Mechanical isolation significantly increased the relative frequency of plasma cells (**Figure 5C**). A similar trend was observed for GC B cells, although the increase did not reach statistical significance (**Figure 5D**).

### 3.6 Mechanical isolation enriches dendritic cells and macrophage subsets

**Figure 6:**
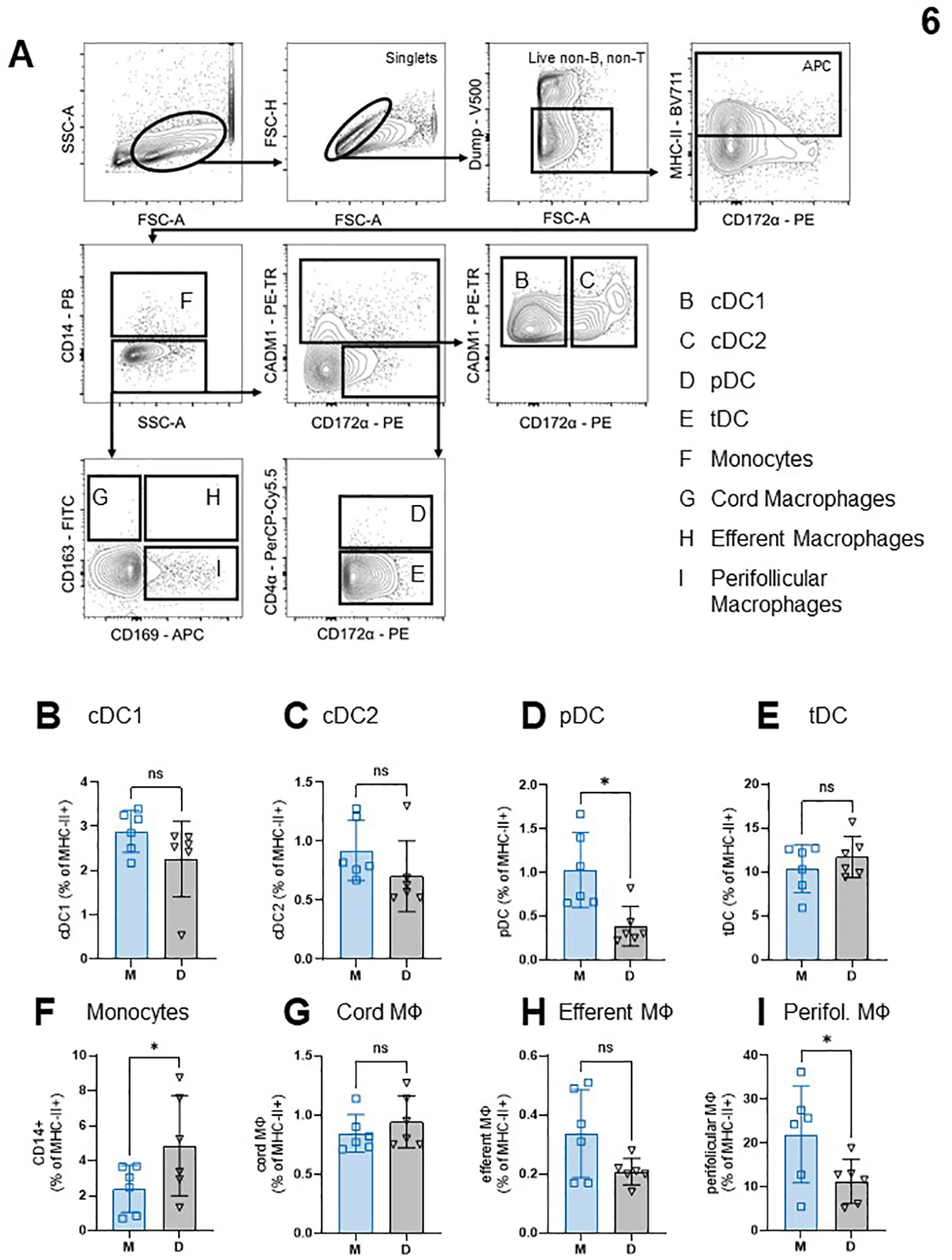
Myeloid cells benefit from mechanical isolation. Gating strategy **(A)** and corresponding frequencies **(B–I)** of conventional dendritic cells type 1 and 2 (cDC1 **(B)**, cDC2 **(C)**), plasmacytoid dendritic cells (pDC **(D)**), transitional dendritic cells (tDC **(E)**), monocytes **(F)** as well as cord **(G)**, efferent **(H)** and perifollicular macrophages **(I)** from MHC-II+, FSC-high, single cells after exclusion of lineage positive and dead cells. Indexed letters **(B–E)** within the flow cytometrical plots indicate corresponding gates and graphs. Data from 6 individual pigs is depicted as mean ± SD, mechanical isolation (M, blue), enzymatic digestion (D, black). Statistical analyses were performed as paired t tests. Significant differences were depicted according to the P-valueas non-significant (ns) (P > 0.05), *(P ≤ 0.05).

Cells of myeloid origin, especially dendritic cells and phagocytes, are an additional critical component of the GCR. Given their pronounced sensitivity to handling, these cells are likely more vulnerable to dissociation−related damage. To evaluate the impact of mechanical isolation versus enzymatic digestion, a pig-specific marker combination was used to identify the dendritic cell subsets cDC1, cDC2, pDC and tDC as previously published (29). Additionally, phagocyte levels were analyzed based on the marker combination of CD14, CD163, and CD169 (**Figure 6A**). Mechanical isolation tended to increase cDC1 and cDC2 frequencies relative to enzymatic digestion (**Figures 6B, C**); however, these differences were not statistically significant. Furthermore, relative frequencies of pDCs were significantly higher in mechanically isolated cells (**Figure 6D**), whereas tDC frequencies were not significantly influenced by dissociation method (**Figure 6E**). Regarding phagocyte frequencies, enzymatic tonsil dissociation significantly enriched CD14+ monocytes compared to mechanical isolation (**Figure 6F**). In turn, mechanical isolation significantly improved the levels of CD14- CD163- CD169+ perifollicular macrophages (**Figure 6I**). A similar pattern, albeit not significant, could be seen for CD14- CD163+ CD169+ efferent macrophages (**Figure 6H**). Relative frequencies of cord macrophages were not significantly affected by isolation procedure (**Figure 6G**).

Furthermore, to evaluate antigen-presentation capacity the mean fluorescence intensity of MHC-II (SLA-II) in all myeloid cells (CD172α+) was compared (**Supplementary Figure 5**). Interestingly, enzymatic digestion increases MHC-II surface expression compared to mechanical isolation, suggesting improved antigen presenting capacity or an alternatively activated phenotype.

Overall, our results suggest that mechanical dissociation supports the isolation of rare myeloid cells when compared to enzymatic digestion. However, enzymatic dissociation may in turn improve antigen presentation capacity.

### 3.7 Enzymatic digestion dampens T cell cytokine responses and reduces aggregate viability

**Figure 7:**
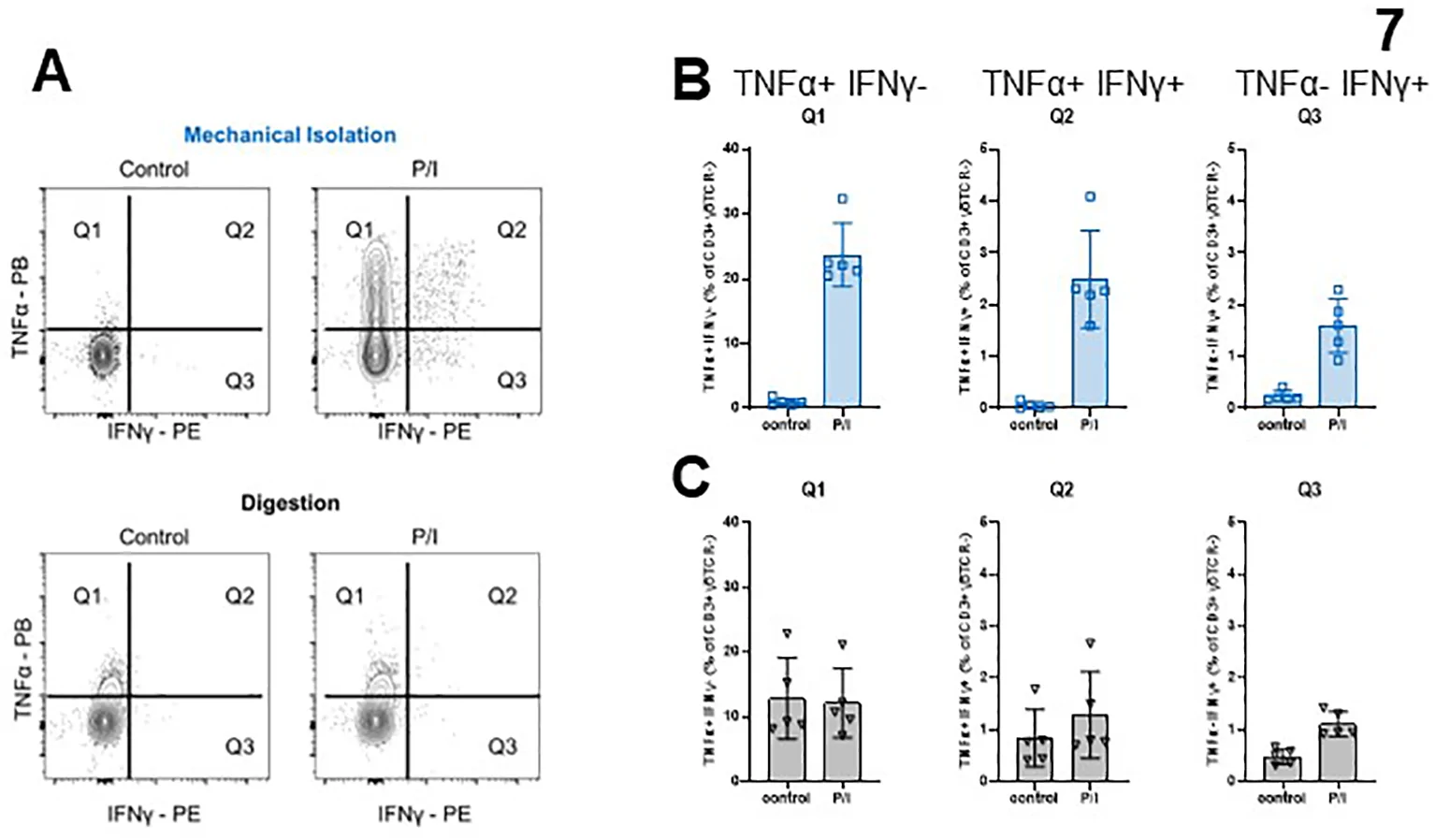
Enzymatic digestion impedes cytokine responses of T cells. **(A)** Representative flow cytometrical blots illustrating the cytokine response of conventional T cells (CD3+γδTCR-). The TNFα and IFNγ response of mechanically **(B)** and enzymatically isolated cells **(C)** to PMA/Ionomycin stimulation was compared. Data from 5 individual pigs is depicted as mean ± SD, mechanical isolation (M, blue), enzymatic digestion (D, black).

Finally, we analyzed how the dissociation method may influence functional properties to determine which isolation method is most appropriate for future GC modelling. First, we assessed the cytokine response of T cells following stimulation with PMA/ionomycin (P/I). Mechanically isolated conventional αβ T cells robustly produced both TNFα and IFNγ upon stimulation (**Figures 7A, B**). Surprisingly, despite showing a higher cell viability, enzymatically isolated T cells were not able to mount similar cytokine responses (**Figures 7A, C**). Notably, even in unstimulated conditions, enzymatic digestion was associated with an elevated baseline cytokine response indicative of their partial activation (**Figures 7A, C**).

**Figure 8:**
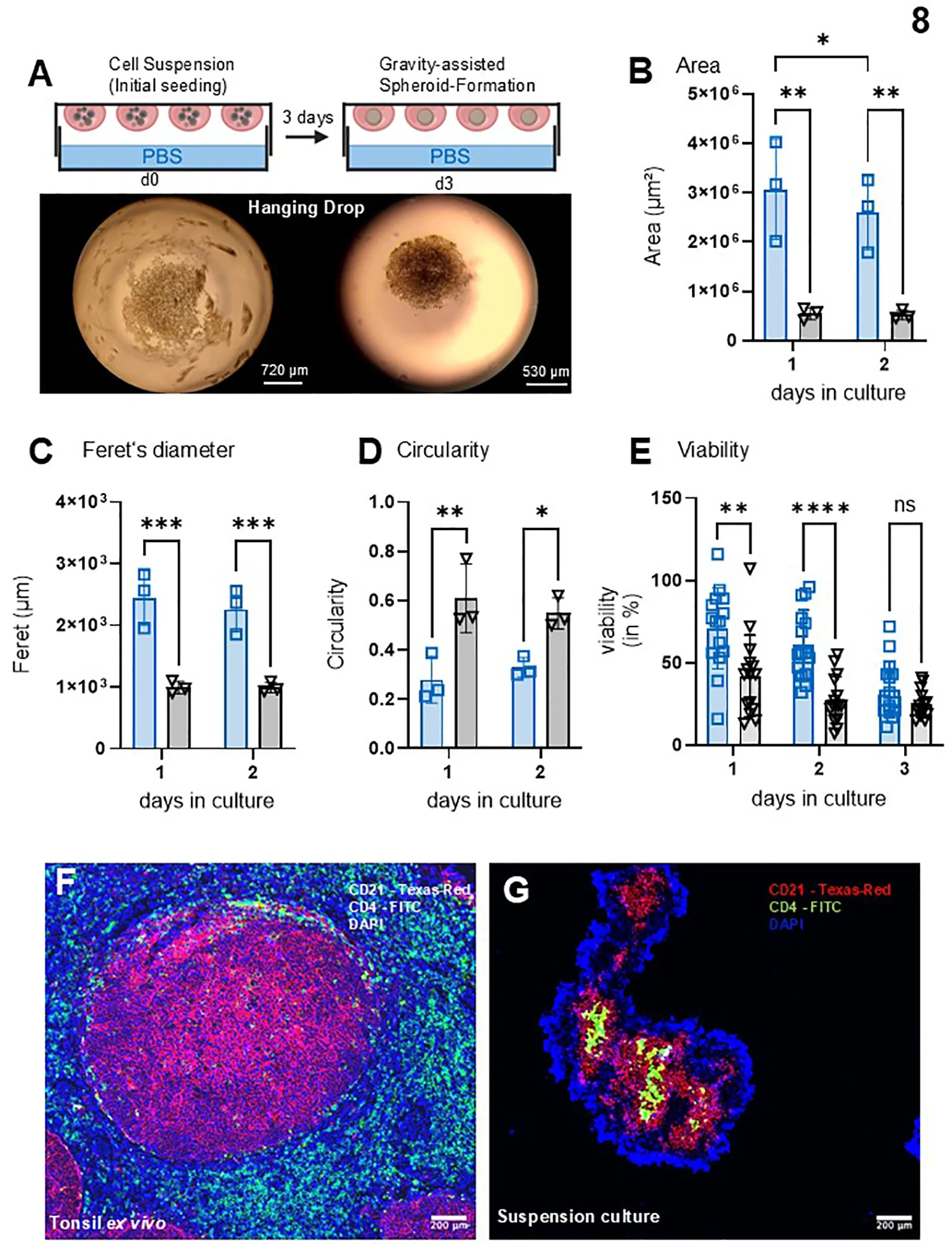
Generation of porcine tonsil-derived spheroids. **(A–E)** Gravity-assisted aggregate-formation via hanging drop method **(A)** was used to form 3D structures from mechanically (left) and enzymatically isolated (right) tonsillar cells. **(B–D)** Morphological analysis of spheroids created with hanging drop aggregation after day 1 and day 2 of incubation Area **(B)**, Feret’s diameter **(C)** and circularity **(D)** were evaluated. Data from three biological replicates, each representing the mean of at least four technical replicates, is depicted as mean ± SD, mechanical isolation (M, blue), enzymatic digestion (D, black). Statistical analysis was performed as paired T-Test. Frequency of viable cells was assessed after up to 3 days of cultivation using the hanging drop method **(E)**. Data from 5 individual pigs (with 3 technical replicates each) is depicted as mean (SD), mechanical isolation (M, blue), enzymatic digestion (D, black). Statistical analysis was performed as two-way ANOVA. **(F, G)** Immunofluorescence imaging of a porcine tonsil with prominent follicles **(F)** and a tonsil-derived 3D culture, generated in suspension culture **(G)** stained with Texas-Red- CD21, FITC-CD4 and DAPI. Significant differences were depicted according to the P-valueas non-significant (ns) (P > 0.05), *(P ≤ 0.05), **(P ≤ 0.01), ***(P ≤ 0.001), ****(P ≤ 0.0001).

Furthermore, we assessed how mechanical versus enzymatic isolation impacted the formation of 3D cultures. For this purpose, we employed both the gravidity assisted hanging drop methos as well as cultivation in suspension culture. Using the hanging-drop method as a gravity-assisted aggregation technique, our data shows that mechanically isolated cells form larger, irregularly shaped spheroids, whereas aggregates formed from digested cells were smaller in size, but more regularly shaped (**Figure 8A**). Quantitative morphological analysis confirmed that mechanically isolated cells indeed produced significantly larger aggregates (day 1: area: 3070193 µm² ± 1010889 µm²; Feret’s diameter: 2447.6 µm ± 447.1 µm) than digested cells (**Figures 8B, C**). However, these spheroids displayed significantly lower circularity, indicating a more angular morphology, compared with those derived from digested cells (circularity, day 1: 0.279 ± 0.095 (M)vs. 0.609 ± 0.139) (**Figure 8D**). Cultivation for up to 3 days further revealed, that 3D cultures with mechanically isolated cells maintained their viability significantly better over time than cultures derived from enzymatically digested tonsils (**Figure 8E**). However, the observed decline in viability in both spheroid types over time indicates that this technique requires further optimization before it can be reliably applied to long−term culture models.

Finally, we investigated whether tonsil spheroids recapitulate follicle structure ex *vivo* by comparing IF imaging of healthy porcine tonsil tissue with that of unstimulated spheroids generated from suspension culture (**Figures 8F, G**). In the tonsillar tissue, a standard follicle structure can be observed; centrally located CD21+ B cells (TexasRed) are surrounded by interfollicular, CD4-CD21-DAPI+ tissue with embedded CD4+ T cells (FITC) (**Figure 8F**). In contrast, in the tonsil spheroids, T cell clusters are surrounded by B cells and other CD4-CD21-DAPI+ cells (**Figure 8G**; **Supplementary Figure 6**). Future research will show whether this inverted pattern is typical for tonsil-derived spheroids.

Taken together, our data suggest that mechanical isolation may be more suitable for functional studies and 3D culture applications, all necessary cellular components of the GC response could be individually detected from mechanically isolated cells. Furthermore, mechanically isolated cells are able to mount a robust cytokine response and maintain viable spheroid structures.

## 4 Discussion

The porcine tonsil represents an important target tissue for investigating mucosal immune responses, vaccine-induced immunity, and germinal center (GC) dynamics. To establish a robust methodological basis for subsequent analyses, we systematically assessed the impact of anatomical sampling site and tissue dissociation strategy on cellular composition, relative frequencies of rare immune subsets, and functional readouts, including cytokine production and the capacity to form 3D cultures in swine tonsillar tissue.

Our study shows that follicle size varies with localization, with larger follicles located in central regions, particularly on the lingual side. While it is known that follicle size and density can vary along mucosal membranes (31), to our knowledge, this is the first direct quantification of tonsillar follicle sizes in regard to anatomical orientation. However, these findings should be interpreted with caution, as only follicle sizes from one animal per orientation were measured. Future studies including multiple individuals per orientation are needed to accurately account for biological variability. Nevertheless, our data indicates that sampling location represents a relevant bias in experimental studies, which should be avoided by standardizing tissue collection e.g. to centrally located, lingual-to-palatine sections.

We focused on comparing mechanical and enzymatic isolation methods in respect to total yield and relative frequencies of specific cell populations. While mechanical dissociation relies solely on physical disruption, enzymatic isolation targets the extracellular matrix of tissue to release cells. The used digestion mix of Collegenase IV, DNAse I, Liberase DH and Liberase TM combines collagenases, proteinases and DNase I to increase cell yields and viability (32). As expected, in our data enzymatic isolation improved the cell viability. However, mechanical isolation yielded significantly more cells than digestion, possibly due to reduced enzymatic stress fragile subsets were better preserved.

Due to limited availability of certain pig genotypes, the lack of on-site lab capacities or to allow for standardization by centralized processing, the shipping fresh samples for extended periods may be inevitable. Therefore, we analyzed how mechanical and enzymatic isolation performed after prolonged storage for up to 2 days. While the total number of isolated cells remained largely stable for mechanically processed samples, viability declined more markedly over time. In contrast, enzymatically digested cells maintained higher viability, indicating that enzymatic digestion may be suitable for shipped samples.

Flow cytometry-based phenotyping revealed that the isolation method had minor impact on the frequencies of conventional αβ and γδ T cells. However, enzymatic digestion significantly enhanced the frequency of rare Tfh cells, including T-bet+Tfh cells. To our knowledge, this study provides the first description of a relatively high frequency of Tfh cells in porcine tonsils. Notably, Tfh cells were detected at higher frequencies in tonsils than in other tissues of pigs of a similar age, like blood and spleen or intestinal lymph nodes (data not shown). This suggests that the tonsil is a valuable source for investigating porcine Tfh biology, especially in the context of the GCR. However, functional assays revealed that enzymatically isolated T cells could not mount robust TNFα and IFNγ responses following stimulation, despite exhibiting a higher viability. Moreover, digestion alone induced elevated baseline cytokine expression in unstimulated samples. These findings suggest to us that enzymatic treatment may induce unspecific activation, as has been described for myeloid cells (33), or interfere with signaling pathways required for subsequent stimulation. Taken together, this indicates that optimal dissociation strategy depends on the downstream application. Enzymatic digestion improved viability and relative frequencies of Tfh and B cell populations, whereas mechanical isolation better preserved T-cell functionality. Therefore, for investigations into functional immune responses of T cells and potentially other GC components, mechanical isolation may be more suitable.

Enzymatic digestion furthermore increased overall B cell frequency, whereas mechanical isolation improved plasma cell frequency. This suggests that B cell subsets may be particularly sensitive to enzymatic digestion. Reportedly, CXCR5 expression is negatively impacted after enzymatic isolation (33), hence, it can be assumed that GCB cells and particularly their interaction with Tfh cells may be implicated (34). Furthermore, there was a trend towards increased germinal center B cell isolation in mechanically isolated samples, indicating that mechanical dissociation may better preserve cellular phenotypes linked to active GCR.

In addition, mechanical isolation significantly increased the relative frequencies of pDCs and perifollicular macrophages, with similar trends observed for cDC1 and cDC2 subsets. In contrast, enzymatic digestion favored monocyte frequency. As myeloid cells are especially delicate and closely interact with their tissue environment, enzymatic digestion may damage or cleave surface molecules or extracellular matrix components, impeding their detection and survival (35). Especially dispase, which is present in Liberase DH (DH= dispase high), is known to affect surface markers (36). Moreover, the prolonged processing time associated with enzymatic digestion may itself contribute to phenotypic changes, together with the known induction of inflammatory pathways following enzymatic isolation (33, 37). Therefore, for studies focusing on interactions between myeloid cells and their cellular environment, mechanical processing may be beneficial. However, our data also shows improved MHC-II expression in enzymatically digested cells (**Supplementary Figure 5**). This could indicate either improved antigen presentation capacity or an altered activation state. Thus, while mechanical isolation may be advantageous for maximizing detection of myeloid subsets, enzymatic activation may influence their phenotypic activation. Furthermore, it has been demonstrated that the transcriptomic signature of enzymatically isolated DCs derived from tonsils and lymph nodes is comparable to blood-derived DCs (38). Therefore, our results are likely linked to the digestion mix used in the study.

The dissociation method also influenced 3D spheroid formation. Mechanically isolated cells formed larger, irregular aggregates that maintained viability longer than those generated from digested cells. However, spheroid architecture did not fully recapitulate native tonsillar follicle organization, suggesting that additional stimuli are required to promote follicle-like self-organization *in vitro* (11). Thus, the current 3D culture system presents a promising platform for further optimization rather than a fully established model of GC reactions. The improved viability and aggregate formation suggest that mechanical isolation may provide favorable conditions for future studies. Furthermore, optimal stimuli for porcine-derived spheroids need to be identified by further research. Translating stimuli and dosages already published for human tonsillar spheroids may prove to be advantageous (10, 11). Moreover, the effects of cryopreservation and long-term cultivation should be assessed to further improve the robustness and applicability of the model.

Currently, the field of porcine GC research is hampered by our inability to identify porcine FDC using commercially available pig-specific monoclonal antibodies. In our hands the previously described cross-reactive CNA.42 antibody (24, 39) failed to detect any specific cell populations. However, it has been hypothesized that antigen retention in GC is less important if antigens are widely available (5). Thus, while the presence of FDC would certainly enhance 3D immunogenicity models, high antigen concentration in combination with other available antigen-presenting cells may compensate for their absence.

Beyond these biological considerations, practical factors may also support the use of mechanical processing in certain experimental settings. Mechanical tonsil dissociation requires less processing time than enzymatic digestion (approximately 60 minutes per 5 samples versus 135 minutes per 5 samples, including 60 minutes of incubation time. In addition, consumable costs were lower for mechanical processing in our experimental setup (**Supplementary Tables 1**, **2**). While these practical aspects were not the primary focus of this study, they may support the implementation of these methods. Moreover, optimization of both dissociation protocols, including enzyme composition and conditions of mechanical and enzymatic processing (i.e. processing time, cooling procedures, media composition), could further improve cell composition and preserve viability and functional integrity. Additionally, our study primarily focused on relative frequencies. Analyzing absolute quantities, particularly for rare cellular subsets such as Tfh or plasma cells, may be beneficial in future studies support our findings and establish functional lymphoid 3D culture models.

Taken together, these findings highlight that selecting a tissue dissociation method requires carefully balancing cell composition, functional integrity, and practical constraints. Neither dissociation method was universally superior, in our experiments. Rather each technique showed distinct advantages depending on the downstream application. Enzymatic digestion improved viability and the detection of Tfh and overall B cells, whereas mechanical dissociation better preserved functional T-cell responses, myeloid cell frequencies, and supported 3D spheroid formation. For studies investigating functional GC-related immune responses, mechanical dissociation may therefore be preferable. Further optimization and functional validation will be required to establish a 3D culture system as an *in vitro* model for GC response.

## Data Availability

The raw data supporting the conclusions of this article will be made available by the authors, without undue reservation.
