# Supplementary material for: Dissociation method influences immune cell composition in porcine tonsil cell suspensions
Source: Front Immunol. 2026 Aug 13;17:1896948. doi: 10.3389/fimmu.2026.1896948 (PMC13518147; doi:10.3389/fimmu.2026.1896948)
Supplement: Supplementary file 1 [file DataSheet1.docx]

# Supplementary Material

Supplementary Table 1: Costs of mechanical isolation based on consumables (as of September 2025)

| **Mechanical isolation (1 sample)** | **price (€)** |
| --- | --- |
| wRPMI (30 mL) | 0,57 € |
| cIMDM (20 mL) | 1,93 € |
| scalpel (1) | 0,26 € |
| strainer (1) | 1,36 € |
| falcon (2) | 0,70 € |
| petridish (1) | 0,23 € |
| transport | 0,48 € |
| **sum** | **5,53 €** |

Supplementary Table 2: Costs of enzymatic isolation based on consumables (as of September 2025)

| **Digestion** | **fix** | **per g of sample** | **per 5 g of sample** |
| --- | --- | --- | --- |
| petri dish (1) | 0,23 € | 0,23 € | 0,23 € |
| falcon (2) | 0,70 € | 0,70 € | 0,70 € |
| strainer (1) | 1,36 € | 1,36 € | 1,36 € |
| digestion mix (1) |  | 6,12 € | 30,59 € |
| cIMDM (60 mL) | 5,80 € | 5,80 € | 5,80 € |
| transport | 0,48 € | 0,48 € | 0,48 € |
| scalpel (1) | 0,26 € | 0,26 € | 0,26 € |
| **sum** |  | **14,95 €** | **39,42 €** |

**Supplementary Figure 1:** Pre-testing for enzymatic digestion. To establish optimal conditions for enzymatic digestion two different enzymatic cocktails were compared (A-B). Additionally, for one cocktail two different pre-cutting procedures were tested (D_1: coarse; D_2: fine). Only cocktail D_3 yielded comparable results to mechanical isolation (MI). This cocktail was subsequently optimized with respect to enzyme concentration and dosing (V1, V2: per volume; D1, D2: per weight; M = mechanical isolation). Furthermore, pre-cutting was further improved by slicing the tissue into 1 mm slices with a scalpel and subsequently macerating it with sharp scissors. Finally, method D2 was selected for all subsequent experiments and is hereafter referred to as “D”.

**Supplementary Figure 2:** FMO controls used for the identification of Tfh cells (A) and Type I Tfh cells (B). According to the gating strategy shown in Figure 4A, Tfh cells were defined as CD3+γδTCR-CD4α+CD45RA-ICOS+Bcl6+ cells. Type I Tfh cells were subsequently identified based on T-bet expression.

**Supplementary Figure 3:** FMO controls used for the identification of B cell subsets. The corresponding gating strategy is shown in Figure 5A. CD16+Blimp-1- negative cells were excluded (A). Plasma cells were identified as Blimp-1+IRF-4+ within the CD3-CD16- population (B). GC-B cells were detected as Pax5+IRF4^low^ cells (C). Lastly, Ki67 expression was assessed (D)

**Supplementary figure 4:** FMO controls used for the identification of cytokine-producing T cells. TNFα and IFNγ expression were assessed within conventional T cells (CD3+ γδTCR-).

**Supplementary Figure 5:** MHC-II (SLA-DR) expression on C172α+ myeloid cells, after exclusion of dead cells, T cells (CD3+, CD8α+) and B cells (CD21+, IgM+) using a dump channel. Representative plots of MHC-II surface expression in mechanically isolated (A) and enzymatically isolated (B) CD172α+ myeloid cells. Mean fluorescence intensity (MFI) of MHC-II is shown as a histogram (C, mechanical isolation blue; enzymatic digestion grey). MHC-II MFI was compared using a paired t-test (D) in six individual pigs. Data is depicted as mean ±SD.

**Supplementary Figure 6:** Individual fluorescence channels and merged image of tonsil spheroids, cultivated in suspension culture showing CD21 (TexasRed, red), CD4 (FITC, green) and DAPI (blue). Scale bar = 200µm
